# Survival and death of intestinal cells infected by *Chlamydia trachomatis*

**DOI:** 10.1371/journal.pone.0215956

**Published:** 2019-04-26

**Authors:** Claudio Foschi, Massimo Bortolotti, Giacomo Marziali, Letizia Polito, Antonella Marangoni, Andrea Bolognesi

**Affiliations:** 1 University of Bologna, Department of Experimental, Diagnostic and Specialty Medicine-DIMES, Microbiology Unit, Bologna, Italy; 2 University of Bologna, Department of Experimental, Diagnostic and Specialty Medicine-DIMES, General Pathology Unit, Bologna, Italy; University of the Pacific, UNITED STATES

## Abstract

The sexually transmitted pathogen *Chlamydia trachomatis* (CT) is able to replicate and survive in human intestinal epithelial cells, being the gastro-intestinal tract a suitable site of residence for this microorganism. In this context, no detailed information about the mechanisms of cell death in intestinal cell lines after a chlamydial infection is available. The aim of this study was to compare the effect of two different CT serovars (D and L2) on the survival/death of different intestinal cell lines (Caco-2 and COLO-205), using endocervical cells (HeLa) as a reference model of genital infection. Seventy two hours after chlamydial infection at different multiplicity of infection (MOI) levels, the viability of HeLa, Caco-2 and COLO 205 cells was evaluated through dose-response experiments by means of a MTS-based assay. To get deeper insights in the mechanisms of cell death induced by CT, cell viability was assessed in presence of different inhibitors (i.e. pan-caspase inhibitor Z-VAD, necroptosis inhibitor Necrostatin-1, hydrogen peroxide scavenger catalase, caspase-1 inhibitor Ac-YVAD-cmk). Moreover, the activation of effector caspases and the presence of cellular apoptotic/necrotic changes were evaluated at different time points after CT infection. Our results demonstrated that, for both chlamydial serovars, intestinal cell lines are more resistant to CT-induced cell death compared to HeLa, thus representing a suitable ‘niche’ for chlamydial residence and replication. In literature, apoptosis has been widely described to be the main cell death mechanism elicited by chlamydia infection. However, our data demonstrate that necroptosis plays a relevant role, proceeding in parallel with apoptosis. The protective effect of catalase suggests the involvement of oxidative stress in triggering both cell death pathways. Moreover, we demonstrated that caspase-1 is involved in CT-induced cell death, potentially contributing to host inflammatory response and tissue damage. Cells infected by L2 serovar displayed a higher activation of effector caspases compared to cells infected with serovar D, suggesting a serovar-specific activation of apoptotic pathways and potentially explaining the greater virulence of L serovars. Finally, we found that *Chlamydia* elicits the early externalization of phosphatidylserine on the external leaflet of plasma membrane independently of caspase activation.

## Introduction

*Chlamydia trachomatis* (CT) is the causative agent of the most common bacterial sexually transmitted infection (STI), worldwide, with a relevant clinical and economic impact [1]. CT serovars from D to K are responsible of common uro-genital infections (i.e. urethritis and cervicitis) and can potentially lead to several sequelae and complications, including pelvic inflammatory disease (PID), tubal infertility and epididymo-orchitis [2]. Notably, CT can be found also at extra-genital sites, as pharyngeal and rectal mucosa, especially in women and ‘men having sex with men’ (MSM) [3]. Specific distinct CT serovars (L1-L3) are associated with lymphogranuloma venereum (LGV), emerging in Europe and North America as a leading cause of proctitis and proctocolitis in MSM, in particular in HIV-positive patients [4].

CT is an obligate intracellular pathogen, able to enter and replicate into different cellular targets, as endocervical and intestinal epithelial cells. During its cycle of development, CT alternates between functionally and morphologically distinct forms: the extracellular, infectious elementary body (EB) and the intracellular, non-infectious, reticulate body (RB). EBs enter the mucosal cells and differentiate into RBs in a membrane bound compartment, called inclusion. CT-containing endosomes avoid fusion with lysosomes and the normal trafficking of intracellular vacuoles is interrupted. After several rounds of replication, RBs start to re-differentiate into EBs and are released from the host cell, ready to infect neighbouring cells [5, 6].

Considering that premature host cell death can limit their replication, chlamydiae are able to activate pro-survival pathways and inhibit apoptosis to guarantee survival within host cells at early and mid-stages (24–48 hours) of intracellular replication [7]. A prominent strategy for preventing cell death includes CPAF mediated degradation of different pro-apoptotic proteins, including BH3-only proteins, Bad, Bim and Puma [7, 8]. In parallel, infection also leads to stabilization of IAP2 (inhibitor of apoptosis protein 2) and up-regulation of the pro-survival factor Mcl-1 (myeloid cell leukemia) [9]. Moreover, CT activates the MAPK and PI3K (phosphoinositide 3-kinase) pathways, eliciting long-lasting survival signals, required for bacterial replication [7]. Finally, CT can also block intrinsic and extrinsic apoptosis, by means of different mechanisms, including the MDM2-mediated ubiquitylation and proteasomal degradation of the tumour suppressor p53 and the inhibition of caspase 8 activation through the master regulator cellular FLICE-like inhibitory protein (cFLIP) [6, 7].

Only at the end of the bacterial developmental cycle (48–72 hours post-chlamydial infection), in a temporally regulated manner, the host cell goes to death. In particular, the release of chamydial EBs can lead to the host cell lysis, a process that results in cell death through permeabilization of the nuclear membrane and calcium-dependent lysis of the plasma membrane [10].

The interaction of CT and host cells in vitro, in term of cell survival and death, has been investigated in different epithelial lines (e.g. HeLa, McCoy, HEp-2) [6, 11, 12], but no exhaustive data are available about the intestinal niche.

Therefore, the aim of this study was to compare the effect of two different CT serovars (D and L2) on the survival/death fate of three cell lines, representing simplified models of endocervical (HeLa cells) and rectal infection (Caco-2 and COLO 205 cells). In particular, investigations about the type of cell death (apoptosis/necrosis) and the mechanisms involved (i.e. protection from cell death by different inhibitors, caspase activation, externalization of phosphatidylserine) were performed.

## Materials and methods

### *Chlamydia trachomatis* strains

Two different CT strains, belonging to the collection of the Microbiology Laboratory of Sant’Orsola-Malpighi Hospital of Bologna (Italy), were used: GO/86, serovar D, and LGV/17, serovar L2. The first strain (GO/86, serovar D) was isolated from a urethral swab of a patient with non-gonococcal urethritis [13], whereas the second one (LGV/16, serovar L2) was recovered from a rectal swab of a male suffering from LGV proctitis. After the isolation, CT strains were propagated for approximately two/three weeks in LLC-MK2 cells (ATCC CCL-7). Afterwards, CT EBs were purified from cell debris by Renografin density gradient centrifugation [14], harvested in sucrose-phosphate-glutamate (SPG) buffer and stored at − 80°C until use. The infectivity titre of each CT strain, expressed as number of inclusion-forming unit (IFUs)/mL, was determined by a serial-dilution method, inoculating suitable dilutions into susceptible cell cultures and calculating the number of inclusions inside the host cells.

### Cell lines

Experiments were conducted using three different epithelial cell lines: HeLa cells (ATCC CCL-2), originated from a human cervix adenocarcinoma, Caco-2 cells (ATCC HTB-37) and COLO 205 cells (ATCC CCL-222), both derived from colorectal adenocarcinoma.

All cell lines were grown in individual tubes containing sterile coverslips (Thermo Fisher Scientific, Waltham, MA) or in 96-well flat-bottom plates in 5% CO_2_ at 37°C, inside a Forma Series II 3110 Water-Jacketed CO2 Incubator (Thermo Fisher Scientific). The cell lines were cultivated in DMEM medium (EuroClone, Pero, Italy), supplemented with 20% foetal bovine serum and 1% L-glutamine, without antibiotics (hereafter referred as ‘complete medium’).

### Cell viability after *C*. *trachomatis* infection

The cytotoxic effect of the different CT serovars (D and L2) was evaluated on endocervical and intestinal cell lines using a MTS [3-(4,5-dimethylthiazol-2-yl)-5-(3-carboxymethoxyphenyl)-2-(4-sulfophenyl)-2Htetrazolium]-based colorimetric assay (CellTiter 96 Aqueous One Solution Cell Proliferation Assay; Promega, Wisconsin, USA). Cells were seeded in a 96-well plate at a density of 1 × 10^4^ cells/well in 100 μL of complete medium and allowed to reach the 60% of confluence. Afterwards, starting from appropriately titrated stock suspensions, cells were infected with purified CT EBs at different levels of multiplicity of infection (MOI 0.1, 0.3, 1, 3 and 10). Finally, cells were centrifuged at 640 × *g* for 2 h and incubated at 37 °C with 5% CO_2_. After 72 h of incubation, the medium was removed from each well and 100 μL/well of medium plus 20 μL/well of colorimetric kit solution were added. After 1 h of incubation at 37°C, the absorbance at 492 nm was measured using a microtiter plate reader Multiskan EX (Thermo Labsystems, Helsinki, Finland). Uninfected cells were used as controls and all the experiments were conducted in triplicate.

In order to confirm CT penetration and to estimate the rate of infection, cells were seeded in parallel in individual tubes containing sterile coverslips in 1 mL of complete medium and infected under the same conditions described above. After 72 h of incubation, CT infection was evaluated by counting chlamydia IFUs by direct immunofluorescence, using a monoclonal antibody against the chlamydial membrane lipopolysaccharide antigen conjugated with fluorescein (Meridian, Cincinnati, OH), as previously reported [14]. The number of IFUs was counted in 30 randomly chosen 200× microscopic fields. Results were expressed as the percentage of infected cells compared to the total number of cells counted.

Direct immunofluorescence was also used to assess the growth kinetics of *C*. *trachomatis* in intestinal cell lines at 24, 48 and 72 hour post-infection.

### Cell viability in presence of inhibitors or scavenger

The cell viability after CT infection was also evaluated on cells pre-treated with 100 μM of the pan-caspase inhibitor Z-Vad-fmk (Z-VAD), 100 μM of the necroptosis inhibitor necrostatin-1 (Nec) and 100 U/ml of the hydrogen peroxide scavenger catalase (CAT) (Sigma Aldrich, Missouri, USA).

To evaluate the involvement of caspase-1 during chlamydial infection, the cell viability was also evaluated on cells pre-treated with 100 μM of the selective caspase-1 inhibitor Ac-YVAD-cmk (Sigma Aldrich).

Cells (1 × 10^4^/well) were seeded in a 96-well plate in 100 μL of complete medium and allowed to reach the 60% of confluence. Inhibitors or scavenger were added 3 h before the infection with CT at MOI 3 and plates were centrifuged at 640 × g for 2 h and incubated at 37 °C with 5% CO_2_. After a 72 h-incubation, cell viability was evaluated as above described.

### Effector caspase activation

Caspase 3/7 activity was assessed through the luminescent assay Caspase-Glo 3/7 (Promega). The kit provides a proluminescent caspase-3/7 DEVD-aminoluciferin substrate and luciferase in a reagent optimized for caspase-3/7 activity, luciferase activity and cell lysis. The substrate cleavage by caspase generates a luminescent signal proportional to the amount of caspase activity. Cells (1 × 10^4^/well) were seeded in 96-well microtiter plates in 100 μL of complete medium. After 24 h, the medium was removed and cells were infected with CT at MOI 3. After incubation at 24, 48 and 72 h, the medium was removed and 50 μL/well of complete medium plus 50 μL/well of Caspase-Glo 3/7 were added. Plates were shaken at 420 rpm for 1 min and then incubated for 20 min at room temperature in the dark. The luminescence was measured by Fluoroskan Ascent FL (Thermo Labsystems), using an integration time of 10 sec. Obtained values were normalized for the percentage of cell viability.

### Flow cytometry analysis

Cells (2 × 10^4^/mL complete medium) were seeded in individual tubes containing sterile coverslips, and after 24 h, they were infected with CT at MOI 3, under the same conditions described above. The experiments conducted in presence of Z-VAD were carried out likewise, pre-treating cells with the inhibitor (100 μM) 3 h before the infection. After 24 or 72 h, cells were trypsinized, centrifuged at 400 × *g* for 5 min, washed in 1 ml of 0.14 M NaCl, 5 mM NaH_2_PO_4_, pH 7 (PBS), centrifuged again in cytofluorimeter tubes (Falcon, Franklin Lakes, NJ, USA) and re-suspended in 294 μL binding buffer provided in the Annexin V-EGFP/PI detection kit (Biovision, Mt. View, CA). Annexin V-EGFP (3 μL) and propidium iodide (PI) (3 μL) were added and tubes were incubated for 10 min in the dark at room temperature.

In flow cytometry, Annexin V is used to detect apoptotic cells by its ability to bind to phosphatidylserine, a marker of apoptosis when it is on the outer leaflet of the plasma membrane. PI is used as DNA stain to evaluate cell viability, considering that it cannot cross the membrane of live cells.

Early-stage apoptotic cells (AnnexinV+/PI−), necrotic cells (AnnexinV−/PI+) and late-stage apoptotic cells (AnnexinV+/PI+) were analysed by flow cytometry within 30 min, using a FACSAria BD analyser using the FACSDiva software (Becton Dickinson and Company, Franklin Lakes, New Jersey, USA), as previously described [15].

### Statistical analysis

Statistical analyses were conducted using XLSTAT-Pro software, version 6.1.9 (Addinsoft 2003) and GraphPad Prism software (GraphPad Prism version 5.02 for Windows, GraphPad Software, San Diego California USA, www.graphpad.com). Results are given as means ± standard deviation (± SD) of three independent experiments. Data were analysed by using t-test or one-way analysis of variance (ANOVA) test, followed by Dunnett’s Multiple Comparison test, where appropriate. Statistical significance was determined at *P* < 0.05 (*), *P* < 0.01 (**) and *P* < 0.0001 (***).

## Results

### Effect of *C*. *trachomatis* infection on cell viability

**Fig 1:**
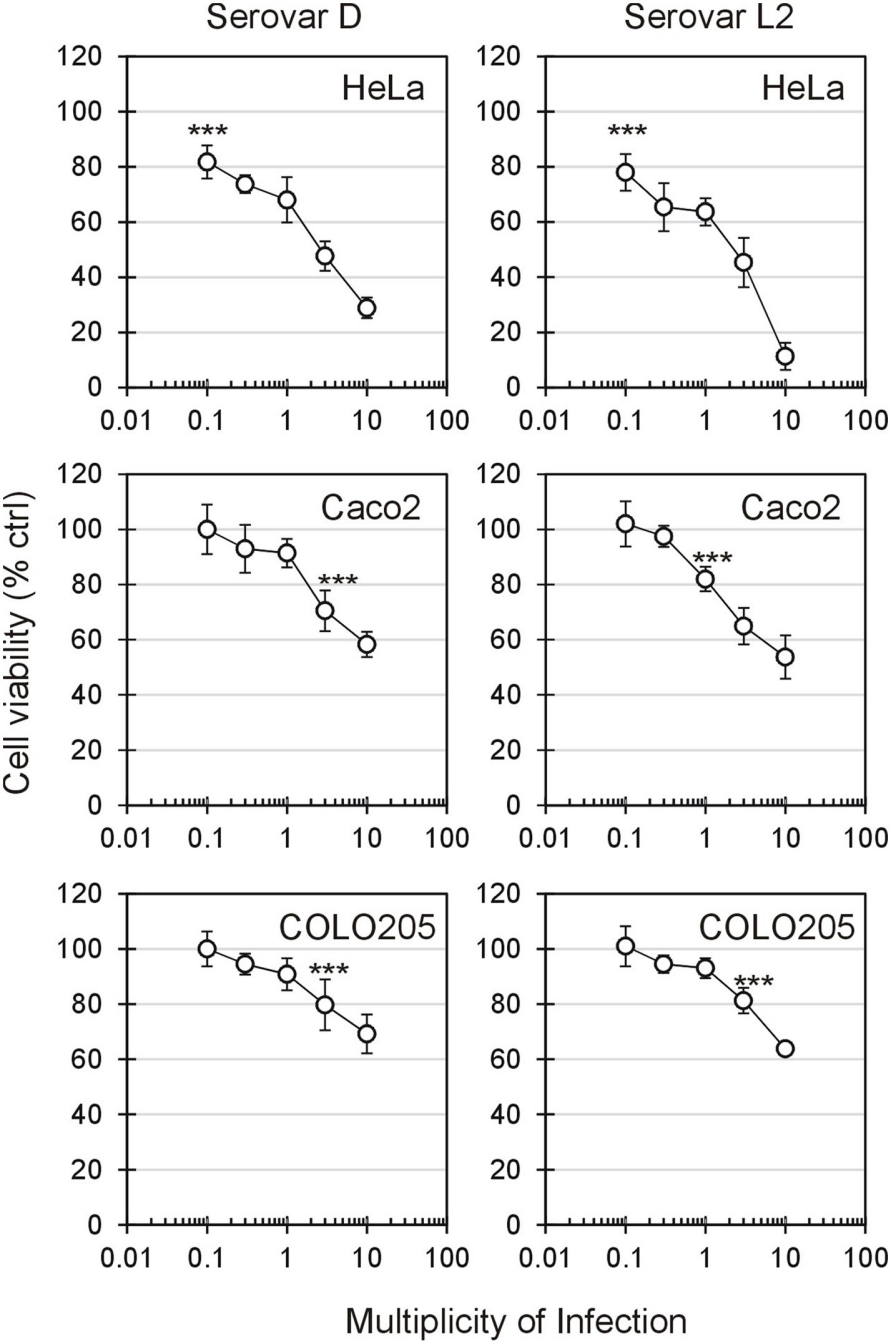
Effect of CT serovar D and L2 on cell viability. Cells were incubated for 72 h with different MOI of CT serovars. Cell viability was measured through MTS salt reduction, as described in the ‘Methods’ section, and expressed as percentage compared to control (uninfected cells), taken as 100%. Results are given as Means ± SD of three independent experiments. SD never exceeded 15%. The asterisks indicate the first MOI at which cell viability significantly decreased (***, *P* < 0.0001) compared to uninfected cells. Statistical analysis was performed by ANOVA test using Dunnett’s multiple comparison.

Seventy two hours after chlamydial infection, the viability of HeLa, Caco-2 and COLO 205 cells was evaluated in dose-response experiments through MTS assay. At equal MOI, the infectivity rate of CT did not differ significantly between the three cell lines and chlamydial serovars (S1 Table). Thus, the sensitivity to chlamydial infection in term of cell death was compared between the different cell lines and CT serovars. As shown in Fig 1, CT reduced cell viability in a dose-dependent manner, but this effect was clearly dependent on the cell origin (endocervical vs intestinal cells). HeLa was the most sensitive cell line: even at a MOI level of 0.1, a significant reduction of cell viability compared to uninfected cells was observed (*P* < 0.0001). Conversely, Caco-2 and COLO 205 cells showed a lower sensitivity to chlamydial infection with respect to HeLa cells. The reduction of cell viability became significant (*P* < 0.0001) at MOI 3 for servovar D and 1 for serovar L2 in Caco-2 cells, and at MOI 3 for both serovars in COLO 205. For HeLa cells, the MOI causing 50% of cell viability reduction after 72 h (Effective MOI 50, EM_50_) was 2.80 for serovar D and 2.03 for serovar L2. Since cell viability was > 50% even at MOI 10, EM_50_ was not calculated for Caco 2 and COLO 205 cells.

Finally, except for COLO 205 cells, we noticed that for various MOI levels (e.g. MOI 1, 3 and 10 in Caco-2 cells and MOI 10 in HeLa), L2 serovar showed a significantly higher (*P* < 0.05) cytotoxic effect compared to D serovar.

Considering that, at MOI 3, the cell viability was significantly reduced in all cell lines, this level of MOI was selected to perform all further experiments.

When evaluating the growth kinetics of *C*. *trachomatis* in intestinal cell lines, for both the serovars and the cell lines included in the study, we observed the typical chlamydial cytoplasmatic inclusions displaying a high degree of granularity that gradually became larger over time (S1 and S2 Figs). Moreover, no small chlamydial inclusions suggestive of persistence (aberrant reticular bodies, with intensely bright inclusions lacking granularity) were found.

### Effect of cell death inhibitors or oxidative stress scavenger on cell viability after CT infection

**Fig 2:**
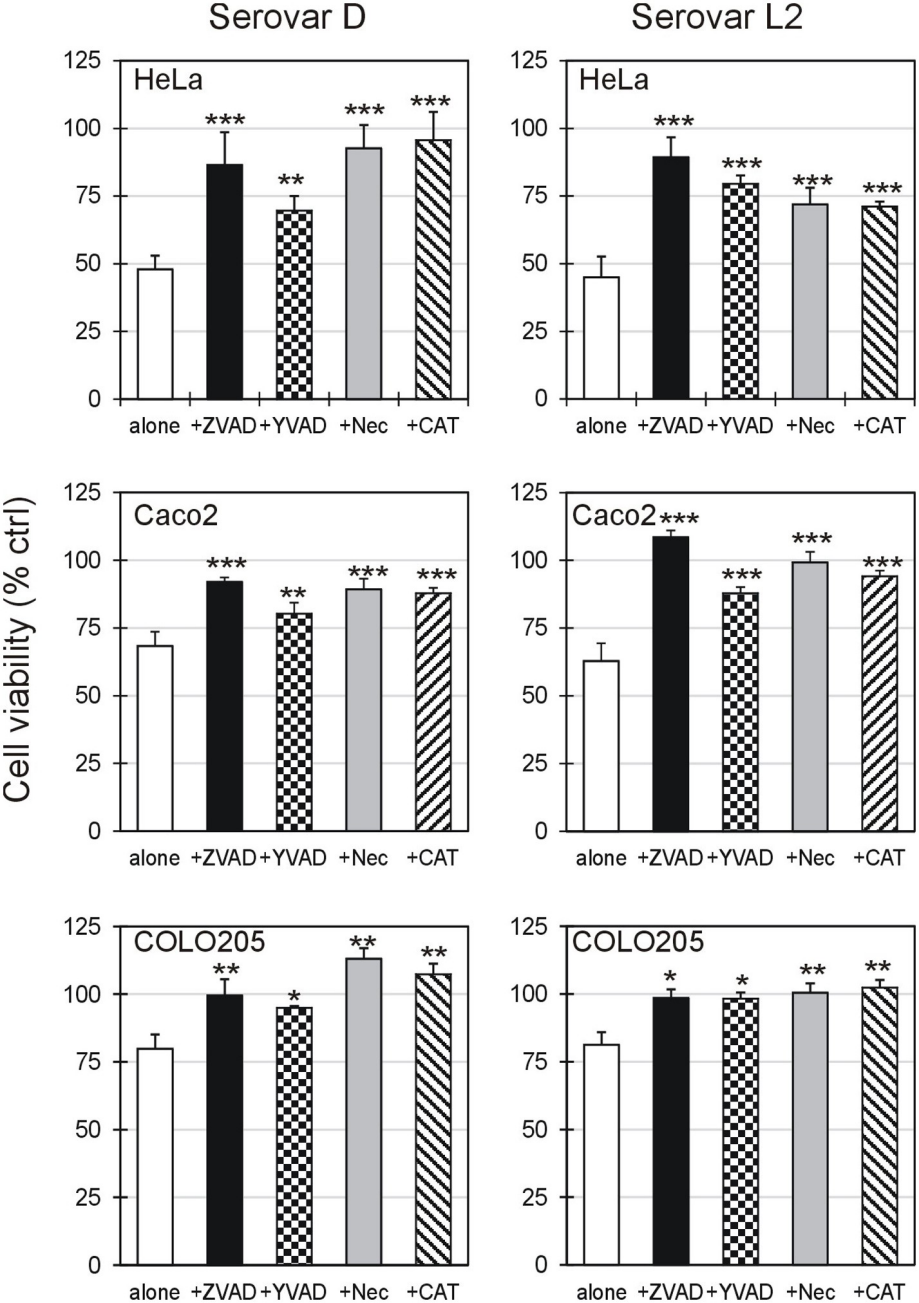
Effect of CT serovar D and L2 on cell viability in presence of cell death/stress inhibitors. Viability of HeLa, Caco-2 and COLO 205 cells 72 h post-chlamydial infection with serovar D (left) and serovar L2 (right) at MOI 3 without (white columns) or in the presence of 100 μM pan-caspase inhibitor (Z-VAD) (black columns), 100 μM caspase 1 inhibitor Ac-YVAD-cmk (YVAD) (checkered columns), 100 μM necroptosis inhibitor necrostatin-1 (Nec) (grey columns) or 100 U/mL hydrogen peroxide scavenger catalase (CAT) (striped columns). Z-VAD, Nec and CAT were added 3 h before CT infection, and the viability was measured after 72 h. Cell viability is expressed as percentage compared to control (uninfected cells), taken as 100%. Results are given as Means ± SD of three independent experiments. Statistical significance was determined by ANOVA test, followed by Dunnett’s multiple comparison. Asterisks indicate significant differences between cells incubated with CT alone and CT plus inhibitors or scavengers in each experimental condition (*** *P* < 0.0001; ** *P* < 0.01; * *P* < 0.05).

In order to evaluate cell death pathways involved in CT infection, two different cell death inhibitors were tested, namely the pan-caspase inhibitor Z-VAD and the necroptosis inhibitor necrostatin-1 (Nec). Additionally, the involvement of oxidative stress was investigated by including the hydrogen peroxide scavenger catalase (CAT). As shown in Fig 2, the pre-treatment with inhibitors or scavenger significantly increased the cell viability for all cell lines and CT serovars, suggesting that caspase-dependent apoptosis, necroptosis and oxidative stress are all involved in cell death mechanisms triggered by CT infection.

Moreover, the pre-treatment with Ac-YVAD-cmk significantly increased the cell viability for all cell lines and CT serovars, indicating the involvement of caspase-1 during the late stages of CT-induced cell death (Fig 2).

### Effector caspase activation after CT infection

**Fig 3:**
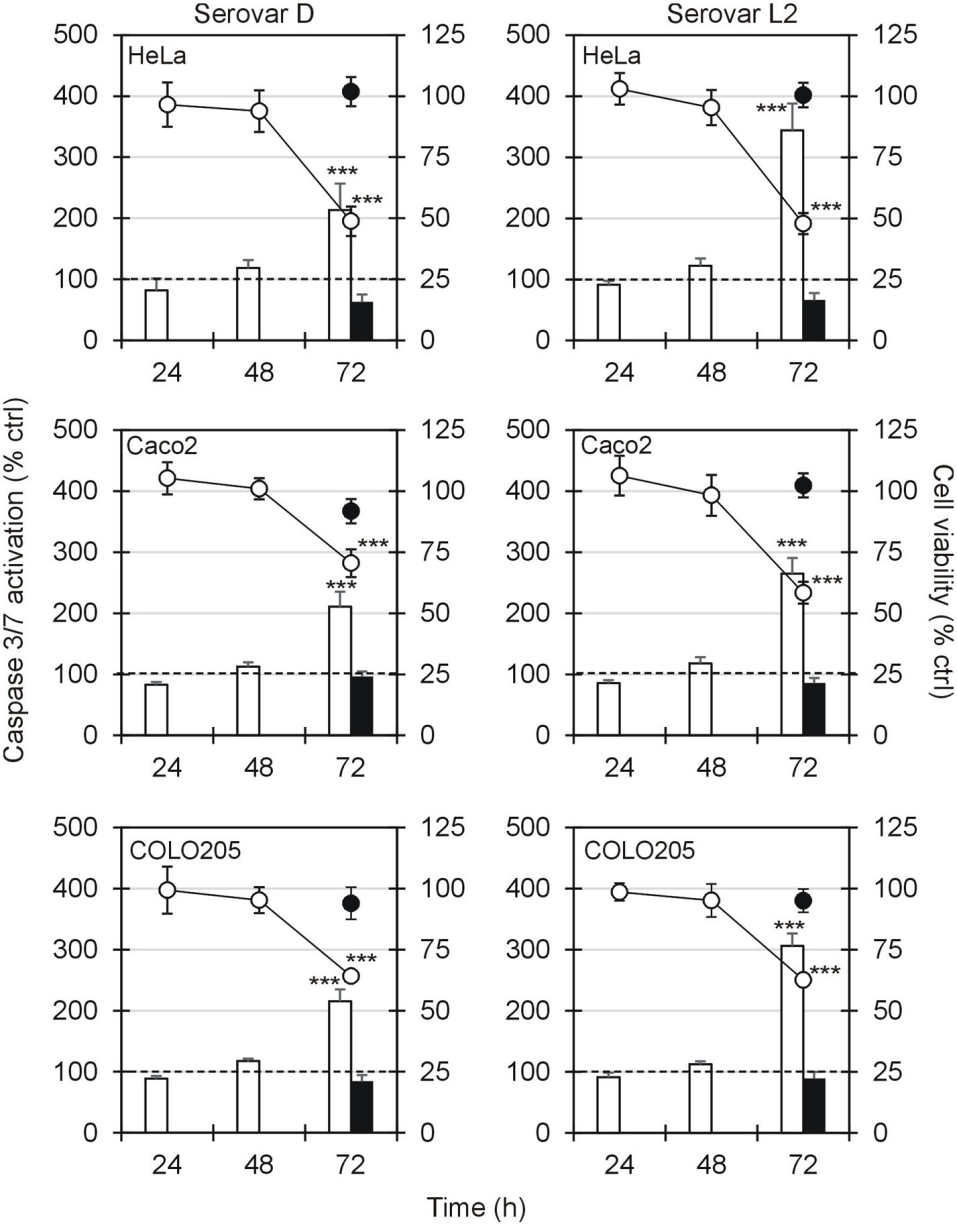
Effector caspase activation after CT infection. The figure shows Caspase 3/7 activation (columns) and cell viability (lines) in HeLa, Caco-2 and COLO 205 cells infected with serovar D or serovar L2 at MOI 3. The activation of caspases was reported as percentage compared to uninfected cell values, taken as 100%. The black column and black single symbol indicate respectively the caspase activation and cell viability in presence of the pan-caspase inhibitor Z-VAD added 3 h before CT infection. Results are given as Means ± SD of three independent experiments. Statistical significance was determined by ANOVA test using Dunnett’s correlation. Asterisks indicate significant differences in each experimental condition between infected and uninfected cells (*** *P* < 0.0001).

The activation of effector caspases 3/7 was measured in cell lines after 24, 48 and 72 h of chlamydial infection with serovar D and L2 at MOI 3 simultaneously to cell viability. As shown in Fig 3, no significant activation of effector caspases was observed after 24 of infection in all cell lines for both serovars. After 48 h, only a slight but not significant increase in caspase activation was observed. On the contrary, a strong caspase 3/7 increase was detected 72 h post-infection, when also cell viability reduction became significant (*P* < 0.0001). Serovar L2 induced a greater activation of effector caspases compared to serovar D (*P* < 0.0001). The pre-treatment with the pan caspase inhibitor Z-VAD caused the complete inhibition of caspase 3/7 activation and the survival of about 100% of CT-infected cells, thus confirming the involvement of caspase-dependent apoptosis (Fig 3).

### Annexin V/PI positivity after CT infection

**Fig 4:**
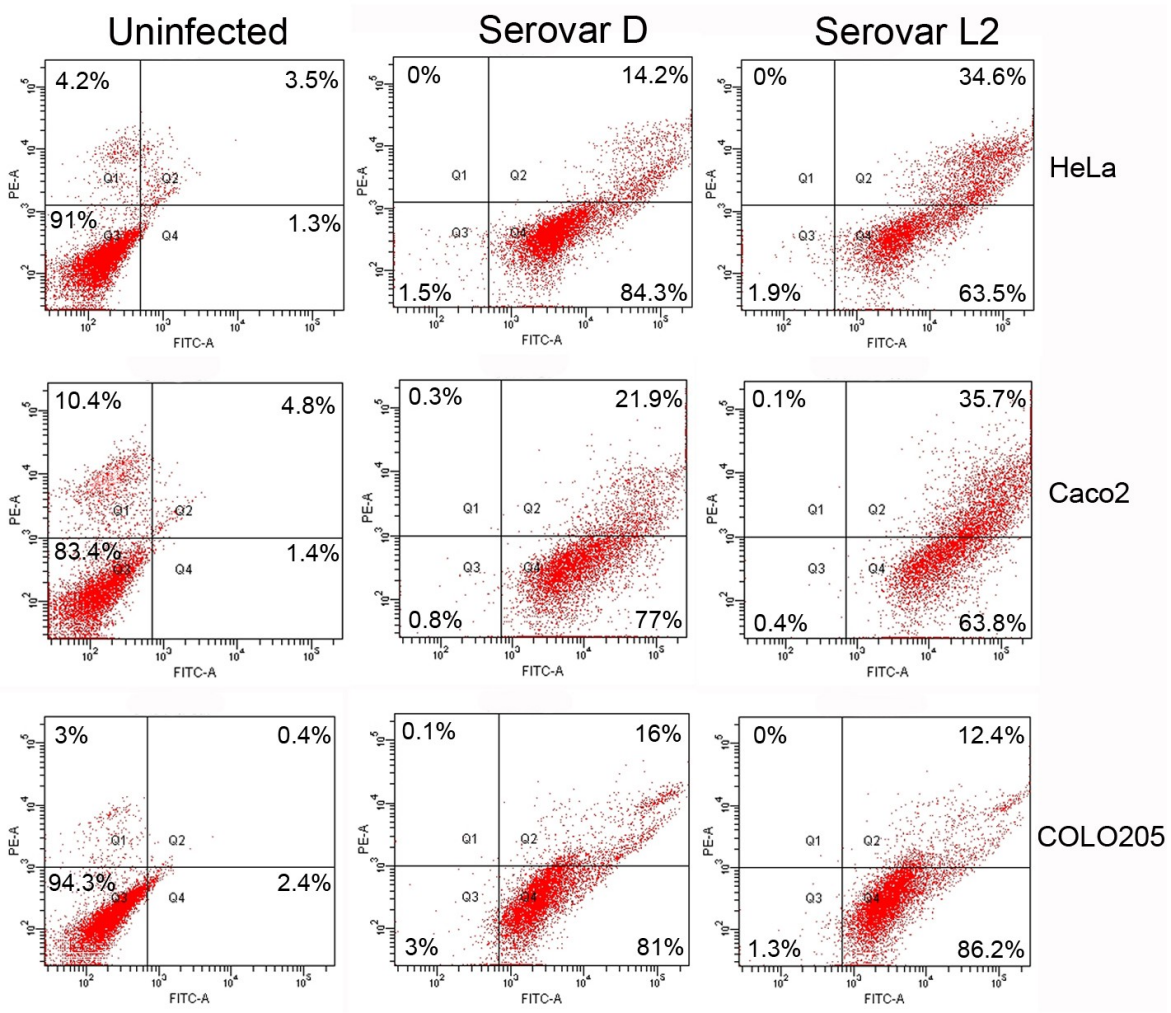
Apoptotic/necrotic changes after CT infection. Cytofluorimetric analysis of Annexin V/propidium iodide double staining of cell lines infected for 72 h with CT serovars D and L2 at MOI 3. FITC-A channel (x-axis) is used for the detection of Annexin V-EGFP fluorescence. PE-A channel (y-axis) is used for the detection of propidium iodide fluorescence. Data are representative of three independent experiments each performed in triplicate.

The presence of membrane apoptotic and necrotic changes in cell lines infected for 72 h by CT (MOI: 3) was evaluated by means of a double staining with Annexin V-EGFP and PI. As shown in Fig 4, for all cell lines and both serovars, more than 90% of infected cells was positive for Annexin V. Most of the cells (65–85%) showed only the positivity for Annexin V (usually indicating early apoptosis), whereas a contemporary detection of Annexin V and PI fluorescence (indicating late apoptosis) was detected for the remaining cells (15–35%). It is worth underlining that less than 0.5% of the cells were positive only for PI, demonstrating the absence of necrosis.

The discrepancy between flow cytometry results (> 90% of cells positive to Annexin V 72 h post chlamydial infection; Fig 4) and the data obtained through MTS assay (40–50% of mortality; Fig 1) led us to evaluate also apoptotic/necrotic changes early after chlamydial infection (i.e. 24 hours) and to assess the effect of Z-VAD on the positivity for Annexin V 72 h post-infection.

As reported in S3 Fig, Annexin V positivity was already present at 24 h post-infection in more than 90% of HeLa and COLO 205 cells and in about 40% of Caco-2 cells. As described in the previous paragraph, at this time, the apoptotic process and the caspase activation induced by *Chlamydia* were still absent, even in presence of an active infection.

Moreover, we found that the positivity for Annexin V was maintained also in presence of the pan-caspase inhibitor Z-VAD (S4 Fig). The maintaining of positivity to Annexin V in presence of Z-VAD, despite the complete caspase inhibition (see Fig 4), confirms that in our experiments phosphatidylserine positivity was not dependent on the apoptotic process, but on the membrane perturbation caused by CT entry into target cells.

## Discussion

In the last years, much attention has been paid to the interaction between *Chlamydia trachomatis* and the gastro-intestinal tract. Animal models of infection and epidemiological studies suggest that the gastrointestinal tract can act as a reservoir of chlamydiae, that can lead to repeat urogenital infections [16–18]. Indeed, it has been shown that human intestinal epithelial cells (Caco-2 cells) support *C*. *trachomatis* replication with a significantly high infectivity [17, 19]. Nevertheless, no detailed information about the mechanisms of cell survival and death in intestinal cell lines after chlamydial infection are available. Thus, we investigated the cytotoxic effect of *C*. *trachomatis* (serovars D and L2) on different human intestinal cell lines (Caco-2 and COLO-205), using endocervical cells (HeLa cells) as a reference model of genital infection.

At first, we found that higher chlamydial amounts are necessary to significantly reduce cell viability in Caco-2 and COLO-205, compared to endocervical cells. Thus, for both chlamydial serovars, intestinal cell lines seem to be more resistant to CT-induced cell death. This aspect is particularly intriguing, if we consider the role of the intestinal tract as a potential reservoir of chlamydiae. The higher survival rate of infected intestinal cells, combined with the good and prolonged chlamydial replication levels, suggest that the intestinal tract could be a suitable site of residence for these microorganisms.

However, these results should be interpreted with caution: cell lines cannot sufficiently mimic the in-vivo pathogen-host interactions, and only the use of more complex and accurate models (e.g. three-dimensional polarized cell line models, in-vivo experiments) will shed light on CT behavior in the colo-rectal site.

Second, we demonstrated that the treatment of cells with different inhibitors of cell death and oxidative stress significantly increases cell viability, rescuing the cells from death after chlamydial exposure. Thus, we found that various mechanisms, such as apoptosis and oxidative stress, are involved in the cytotoxic effect of *C*. *trachomatis* infection in intestinal cells, in agreement with what have been previously described for cell lines of different origin [6, 20–22].

It has been shown that *C*. *trachomatis* induced a transient increase in the reactive oxygen species (ROS) level in epithelial cells within a few hours from the infection, followed by a rapid return to basal level [23]. In parallel, the treatment of infected cells with the antioxidants ascorbic acid and alpha-tocopherol reduces the degree of cell death, suggesting that the redox state of the cell is a regulator in chlamydia-induced apoptosis [11]. On the basis of our data, it is not possible to speculate about the exact role of the oxidative stress in *C*. *trachomatis*-infected cells and further studies will be needed to evaluate the involvement of ROS production in the mechanisms of cell death.

Our data show that other complementary mechanisms, as necroptosis, can play a role in the host cell death induced by CT. Indeed, we found that the inhibition of the RIPK-1 kinase, essential to start necroptosis process, significantly reduces the mortality of infected cells. Necroptosis is a form of regulated cell death, which can be initiated by a variety of triggers, including TNF, interferon, bacterial lipopolysaccharide, dsRNA, viral infection and anti-cancer drugs [24, 25]. We can speculate that, as for other mechanisms of cellular ‘suicide’, the epithelial cells, through the activation of necroptosis, can prevent the completion of pathogen replication cycles, thus blocking the progression of chlamydial infection. On the other hand, necroptosis can also promote IL-1β production and inflammation [26], thus potentially participating to the tissue damage in case of persistent and chronic *C*. *trachomatis* infections.

Similarly, the involvement of caspase-1 in CT-induced cell death goes in the same direction. Indeed, caspase-1, activated in response to infection by a macromolecular protein complex termed ‘inflammasome’, is able to cleave pro-IL-1β into its mature form, contributing to host inflammatory response and tissue damage [27, 28].

Afterwards, in order to get deeper insights in the mechanisms of cell death, we evaluated apoptotic and necrotic changes through flow cytometry (Annexin V/PI analysis) and caspase 3/7 activation, at different time points after CT infection.

We confirmed that apoptosis is a crucial mechanism of epithelial cell death after a chlamydial infection, irrespective of CT serovar and cell line origin [6, 21, 22]. This process, already known as ‘chlamydial paradox’ [11], was clearly linked to the activation of effector caspases (caspases 3/7) and it was evident only 72 hour post-infection, being still absent at early and mid-stages (24–48 hours) of chlamydial intracellular replication [29, 30].

As already mentioned in the results section, it should be taken into account that the membrane disturbance due to the CT entry into the cell induces Annexin V positivity. In this context, considering that cytometry analysis cannot distinguish between cells in late apoptosis and cells in necroptosis (positive for Annexin V and propidium iodide in both cases), the exact contribution of the two different death pathways remains unclear, making further investigations necessary.

Other interesting data emerged from the comparison between the two chlamydial serovars: cells infected by chlamydial L2 serovar displayed a higher activation of effector caspases compared to cells infected with serovar D. This aspect could be potentially ascribed to a different and serovar-specific activation of apoptotic pathways. Moreover, this phenomenon could explain one of the reasons of the greater virulence and pathogenic capacity displayed by chlamydial L serovars during genital and extra-genital infections [4, 31]. In agreement with these data we also found that globally L serovar showed a higher lethal effect than D serovar at least in Caco-2 and HeLa cells.

Finally, the apparent discrepancy of the mortality rate between MTS assay (40–50%) and flow cytometry (> 90%) prompted us to evaluate the effect of CT at only 24 hours post-infection and the effect of a broad-spectrum caspases inhibitor (Z-VAD).

We highlighted that at early stages post-infection (24 hours), in absence of caspase activation, CT induces the externalization of the lipid phosphatidylserine on the surface of epithelial cells. In addition, at 72 hours post-infection, Z-VAD was able to reduce the number of cells in late apoptosis (Annexin V +/ PI +), but it had no effect on cells expressing only phosphatidylserine (Annexin V +/ PI -).

The immediate, caspase-independent, externalization of phosphatidylserine has been previously shown for various cell lines [32], but here, for the first time, this phenomenon was demonstrated for intestinal cells. Phosphatidylserine exposure is an early marker of apoptosis and it is associated with the proinflammatory triggering of the alternative pathway of complement activation [32, 33]. In agreement with Ojcius *et al*. [34], we found that the appearance of apoptosis markers in CT-infected cells are not attenuated by a pan-caspase inhibitor. Thus, probably, the involvement of caspases in the externalization of phosphatidylserine elicited by chlamydia, is absent or at least, very limited. In this regard, it should be taken into account that the host lipids trafficking is severely affected by chlamydia infection, as already demonstrated on genital-derived cell lines [35].

It is worth mentioning that, in vivo, the externalization of phosphatidylserine represents a key ‘eat me’ signal for the engulfment of apoptotic cells by phagocytes [36]. However, especially L chlamydial serovars can survive from intracellular elimination in macrophages, using them as Trojan horses for bacterial dissemination [37, 38].

In conclusion, the major findings of this work are the following: (i) intestinal cell lines show a significant resistance to CT-induced cell death, representing a suitable ‘niche’ for chlamydial residence and replication; (ii) beside apoptosis, necroptosis plays a relevant role and the protective effect of catalase suggests the involvement of oxidative stress in triggering both cell death pathways; (iii) caspase-1 is involved in CT-induced cell death, potentially contributing to host inflammatory response and tissue damage; (iv) early after the infection, in absence of a caspase-activation, CT elicits the externalization of the lipid phosphatidylserine on the surface of epithelial cells.

Our results have added knowledge to the pathogenic mechanisms of chlamydia infection in intestinal cells, but further studies are needed to better understand the molecular pathways involved in the cell damage consequent to CT internalization and to better define the role of the intestinal cells in the pathogenesis of chlamydial infections.

## Supporting information

S1 Table Infectivity rate of *C*. *trachomatis* serovars (D and L2) at the different MOI in the various cell lines (HeLa Caco-2, COLO 205). Results are expressed as the percentage of infected cells compared to the total number of cells counted. MOI: multiplicity of infection.
(DOCX)

S1 Fig Growth kinetics of *C*. *trachomatis* in Caco-2 cells. Cells infected with serovar D and L2 were stained with a monoclonal antibody against the chlamydial membrane lipopolysaccharide antigen conjugated with fluorescein. The morphology of the chlamydial inclusions were evaluated at 24, 48 and 72 hours post-infection. Magnification 200×.
(JPG)

S2 Fig Growth kinetics of *C*. *trachomatis* in COLO 205 cells. Cells infected with serovar D and L2 were stained with a monoclonal antibody against the chlamydial membrane lipopolysaccharide antigen conjugated with fluorescein. The morphology of the chlamydial inclusions were evaluated at 24, 48 and 72 hours post-infection. Magnification 200×.
(JPG)

S3 Fig Cytofluorimetric analysis of Annexin V staining of cell lines infected for 24 h with CT serovars D and L2 at MOI 3. FITC-A channel (x-axis) is used for the detection of Annexin V-EGFP fluorescence.
(JPG)

S4 Fig Cytofluorimetric analysis of Annexin V/propidium iodide double staining of cell lines infected for 72 h with CT serovars D and L2 at MOI 3 in presence (100 μM) or in absence of the pan-caspase inhibitor Z-VAD. Bars represent the percentage of cells that are Annexin V +/ PI–(up) and Annexin V +/ PI + (down).
(JPG)
