# Supplementary material for: Survival and death of intestinal cells infected by Chlamydia trachomatis
Source: PLoS One. 2019 Apr 26;14(4):e0215956. doi: 10.1371/journal.pone.0215956 (PMC6485707; doi:10.1371/journal.pone.0215956)
Supplement: S1 Table — Results are expressed as the percentage of infected cells compared to the total number of cells counted. MOI: multiplicity of infection. (DOCX) [file pone.0215956.s001.docx]

|  | **HeLa, Caco-2 and COLO 205 cells** | | | | |
| --- | --- | --- | --- | --- | --- |
|  | **MOI: 0.1** | **MOI: 0.3** | **MOI: 1** | **MOI: 3** | **MOI: 10** |
| **CT serovar D** | 25-30% | 35-40% | 65-70% | 85-90% | 100% |
| **CT serovar L2** | 25-30% | 35-40% | 65-70% | 85-90% | 100% |
