# Supplementary figures and images for: Survival and death of intestinal cells infected by Chlamydia trachomatis
Source: PLoS One. 2019 Apr 26;14(4):e0215956. doi: 10.1371/journal.pone.0215956 (PMC6485707; doi:10.1371/journal.pone.0215956)

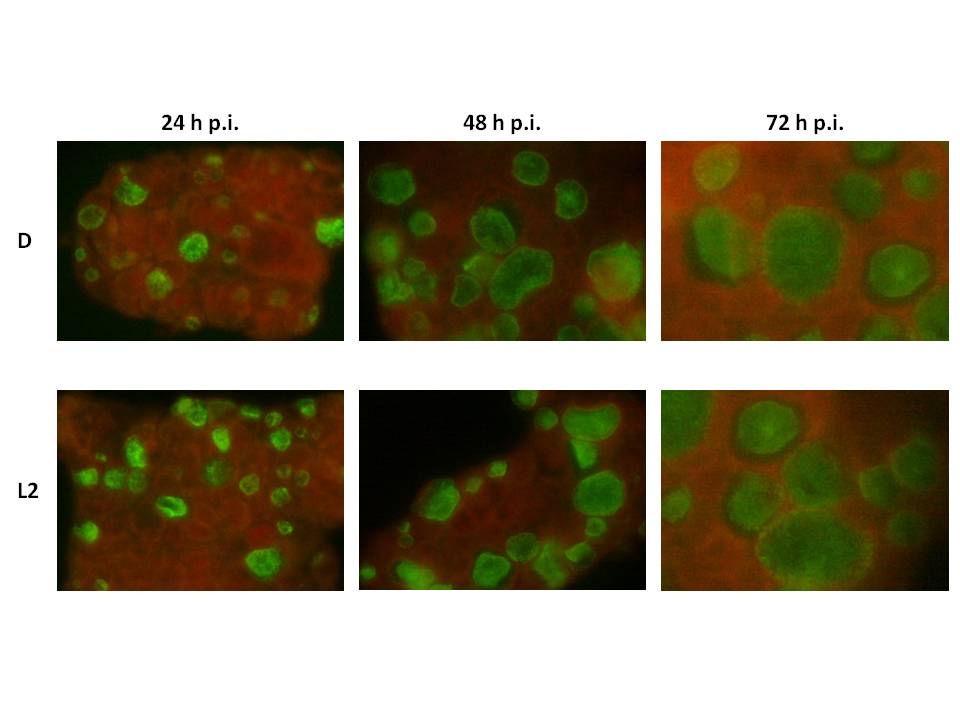

Supplement: S1 Fig — Cells infected with serovar D and L2 were stained with a monoclonal antibody against the chlamydial membrane lipopolysaccharide antigen conjugated with fluorescein. The morphology of the chlamydial inclusions were evaluated at 24, 48 and 72 hours post-infection. Magnification 200×. (JPG) [file pone.0215956.s002.jpg]

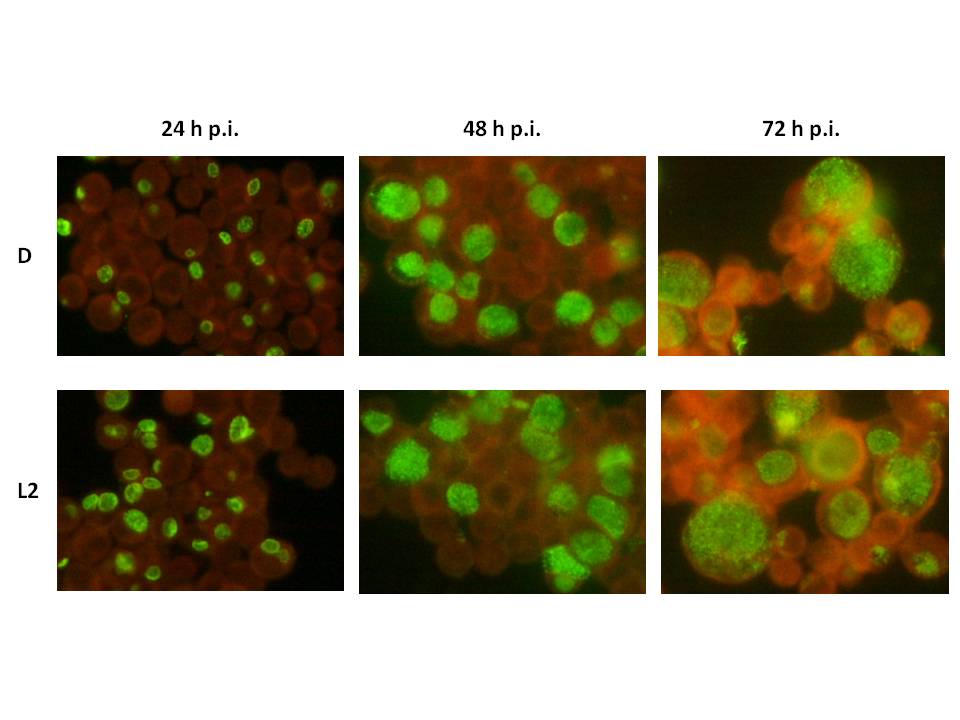

Supplement: S2 Fig — Cells infected with serovar D and L2 were stained with a monoclonal antibody against the chlamydial membrane lipopolysaccharide antigen conjugated with fluorescein. The morphology of the chlamydial inclusions were evaluated at 24, 48 and 72 hours post-infection. Magnification 200×. (JPG) [file pone.0215956.s003.jpg]

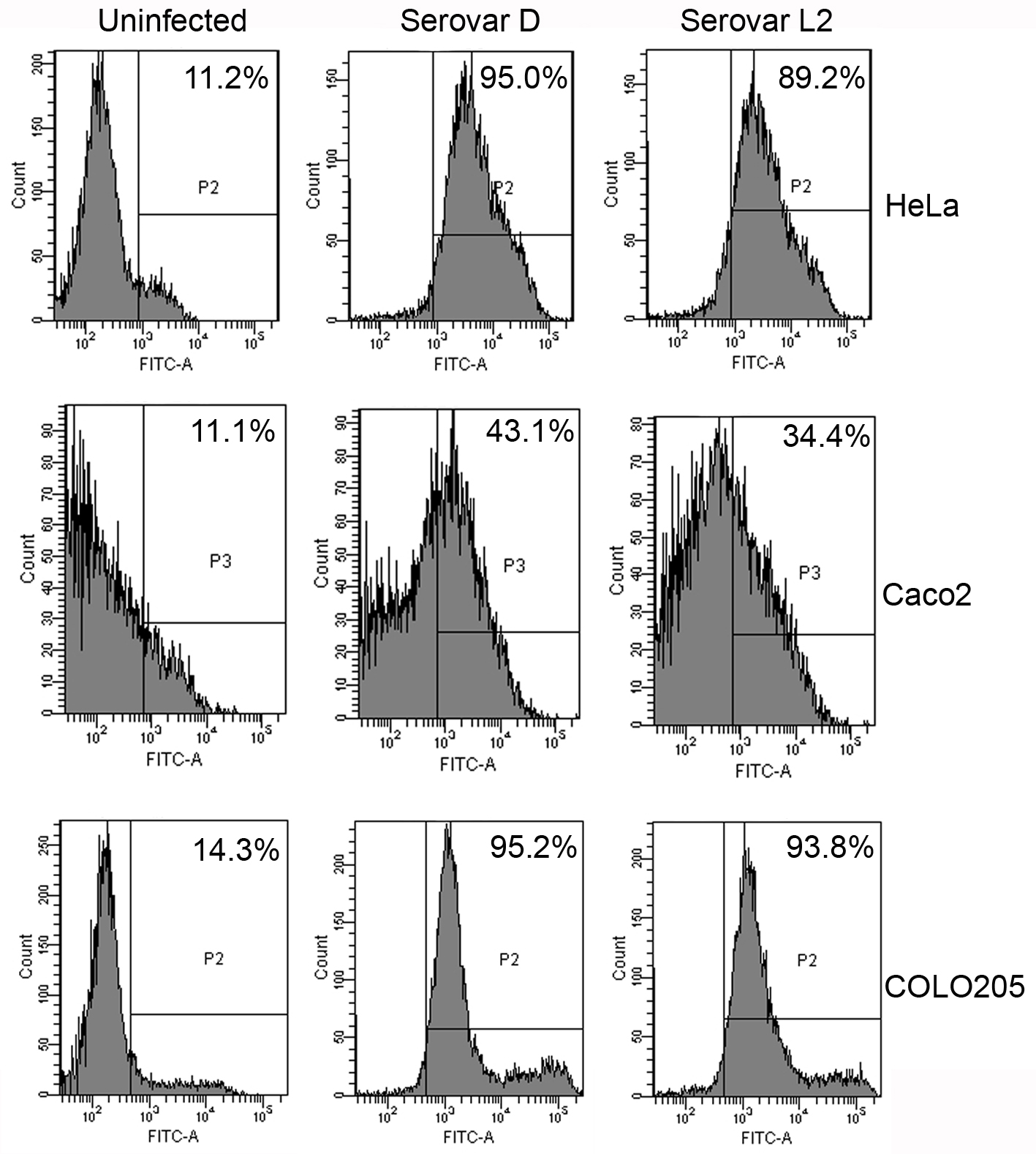

Supplement: S3 Fig — FITC-A channel (x-axis) is used for the detection of Annexin V-EGFP fluorescence. (JPG) [file pone.0215956.s004.jpg]

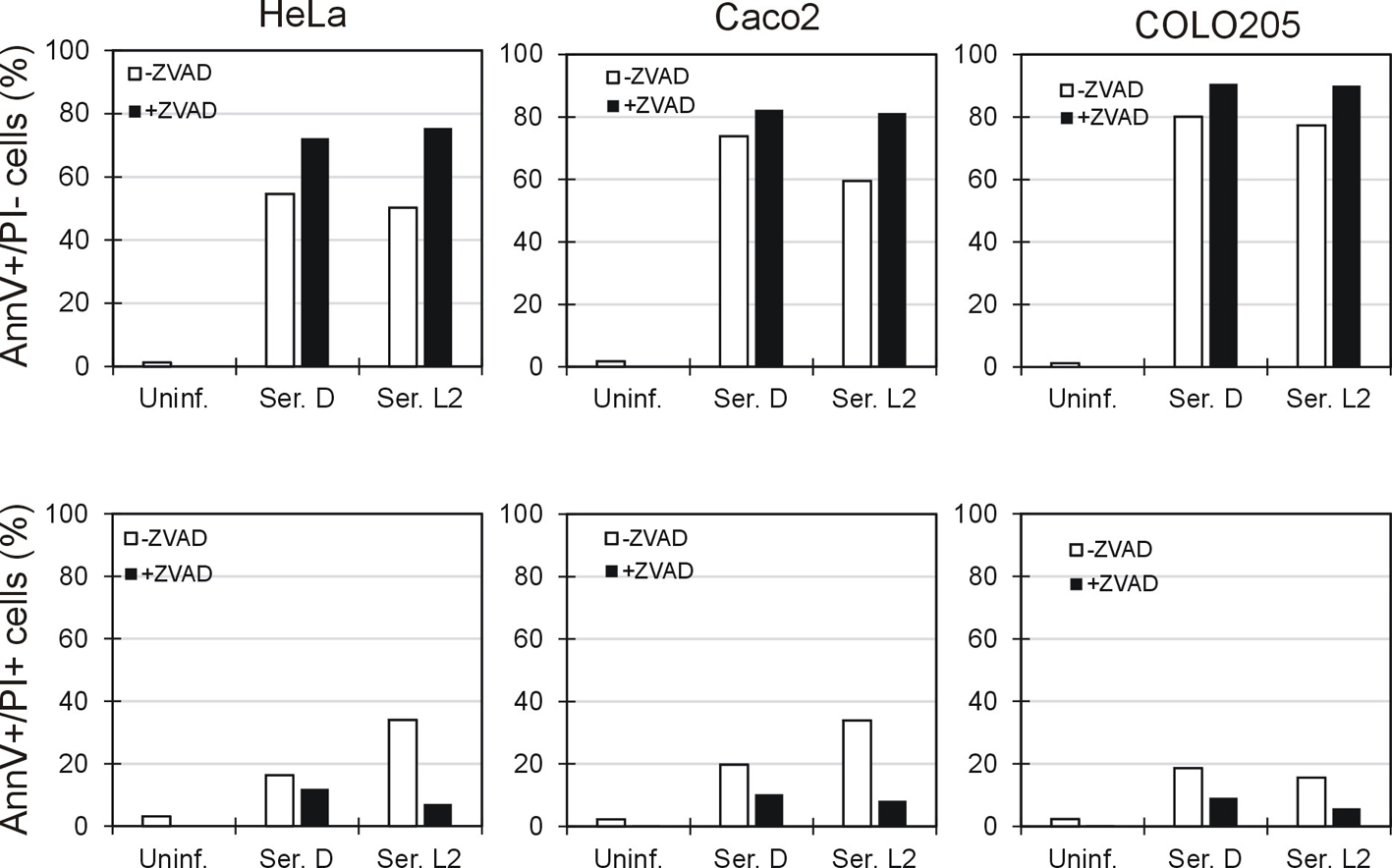

Supplement: S4 Fig — Bars represent the percentage of cells that are Annexin V +/ PI–(up) and Annexin V +/ PI + (down). (JPG) [file pone.0215956.s005.jpg]
